# Diagnostic and Therapeutic Management of Mesothelioma of the Tunica Vaginalis Testis: A Population-Based Study in Italy

**DOI:** 10.3390/cancers17193249

**Published:** 2025-10-07

**Authors:** Giovanni Luca Ceresoli, Simona Stella, Barbara Dallari, Riccardo Perduri, Cinzia Storchi, Luigi Vimercati, Sara Piro, Lucia Giovannetti, Ugo Fedeli, Veronica Casotto, Enrica Migliore, Antonella Stura, Carlo Genova, Lucia Benfatto, Francesca Larese Filon, Flavia D’Agostin, Ilaria Cozzi, Italo Francesco Angelillo, Eugenia Spata, Stefano Murano, Iolanda Grappasonni, Cristiana Pascucci, Massimo Melis, Fabrizio Stracci, Alessandro Marinaccio, Alessandra Binazzi, Dario Consonni, Carolina Mensi

**Affiliations:** 1 Department of Medical Oncology, Humanitas Gavazzeni Clinic, 24125 Bergamo, Italy; giovanniluca.ceresoli@gmail.com; 2 COR Lombardia, Occupational Health Unit, Fondazione IRCCS Ca’ Granda Ospedale Maggiore Policlinico, 20122 Milan, Italy; simona.stella@policlinico.mi.it (S.S.); barbara.dallari@policlinico.mi.it (B.D.); dario.consonni@policlinico.mi.it (D.C.); 3 COR Mesoteliomi Emilia Romagna, AUSL-IRCCS Reggio Emilia, 42123 Reggio Emilia, Italy; riccardo.perduri@ausl.re.it (R.P.); cinzia.storchi@ausl.re.it (C.S.); 4 COR Puglia, Section of Occupational Medicine “B. Ramazzini”, Department of Interdisciplinary Medicine, University of Bari Aldo Moro, 70121 Bari, Italy; luigi.vimercati@uniba.it; 5 COR Toscana, Cancer Risk Factors and Lifestyle Epidemiology Unit, Institute for Cancer Research, Prevention and Clinical Network (ISPRO), 50139 Firenze, Italy; s.piro@ispro.toscana.it (S.P.); l.giovannetti@ispro.toscana.it (L.G.); 6 COR Veneto, Azienda Zero, 35131 Padova, Italy; ugo.fedeli@azero.veneto.it (U.F.); veronica.casotto@azero.veneto.it (V.C.); 7 COR Piemonte, Cancer Epidemiology Unit, CPO and University of Turin, 10124 Torino, Italy; enrica.migliore@cpo.it (E.M.); antonella.stura@cpo.it (A.S.); 8 COR Liguria, Dipartimento di Medicina Interna e Specialità Mediche, Università Degli Studi di Genova and UO Clinica di Oncologia Medica, IRCCS Ospedale Policlinico San Martino, 16132 Genova, Italy; carlo.genova@hsanmartino.it (C.G.); lucia.benfatto@hsanmartino.it (L.B.); 9 COR Friuli Venezia Giulia, Clinical Unit of Occupational Medicine, University of Trieste, 34127 Trieste, Italy; larese@units.it (F.L.F.); fladagostin@yahoo.it (F.D.); 10 COR Lazio, Department of Epidemiology, Lazio Regional Health Service, ASL Roma 1, 00139 Rome, Italy; i.cozzi@deplazio.it; 11 COR Campania, Dipartimento di Medicina Sperimentale, Università degli Studi della Campania “Luigi Vanvitelli”, 80138 Napoli, Italy; italof.angelillo@unicampania.it; 12 COR Sicilia, Cancer Registry ASP Ragusa and Sicily Regional Epidemiological Observatory, 97100 Ragusa, Italy; eugenia.spata@asp.rg.it; 13 COR Bolzano, Azienda Sanitaria dell’Alto Adige, 39100 Bolzano, Italy; stefano.murano@sabes.it; 14 COR Marche, School of Medicinal and Health Products Sciences, University of Camerino, 62032 Camerino, Italy; iolanda.grappasonni@unicam.it (I.G.); cristiana.pascucci@unicam.it (C.P.); 15 COR Sardegna, Regional Epidemiological Observatory, 09123 Cagliari, Italy; massimo.melis@aslcagliari.it; 16 COR Umbria, Section of Public Health, Department of Medicine and Surgery, University of Perugia, 06123 Perugia, Italy; fabrizio.stracci@unipg.it; 17 DIMEILA, Department of Occupational and Environmental Medicine, Epidemiology, Hygiene, National Institute for Insurance against Accidents at Work (INAIL), 00144 Rome, Italy; a.marinaccio@inail.it (A.M.); a.binazzi@inail.it (A.B.)

**Keywords:** mesothelioma, tunica vaginalis testis, rare cancers, treatment, survival

## Abstract

**Simple Summary:**

Mesothelioma of the tunica vaginalis testis (MTVT) is an extremely rare tumor, for which only case reports, small case series and case reviews have been published. To our knowledge, this is the largest population-based study on MTVT, including rigorous assessment of asbestos exposure and a full review of medical records with detailed clinical data on diagnosis and treatment. This study was based on data extraction from the Italian Mesothelioma Registry (Registro Nazionale Mesoteliomi, ReNaM) dataset and confirmed the extreme rarity of MTVT, with a crude incidence rate in 1994–2021 of 0.17 per million person-years in Italy. Epidemiological characteristics of the disease included late age onset, prevalent epithelioid histology and relationship with predominantly occupational exposure to asbestos. Overall median survival was 26.2 months. Surgery was confirmed as the cornerstone of treatment of MTVT, however the optimal extent of resection and the role of adjuvant treatments remain undefined.

**Abstract:**

Background: Mesothelioma of the tunica vaginalis testis (MTVT) is an exceedingly rare tumor. We performed a registry-based study on MTVT patient management and survival in Italy. Methods: Cases were extracted from the dataset of the Italian National Mesothelioma Registry. A descriptive analysis of patient characteristics, including asbestos exposure, clinical presentation, diagnostic work-up and therapeutic management, was performed. Overall survival was evaluated. We calculated hazard ratios (HR) and 95% confidence intervals (CI) for selected variables by fitting univariate and multivariable Cox models. Results: Overall, 104 patients with MTVT were included. Median age was 72 years (range 17–92). Epithelioid histotype was the most frequent. Previous asbestos exposure was identified in two thirds of cases. Data on diagnostic and therapeutic management were available for 74 patients (71%). The most frequent presentations were scrotal swelling/mass, hydrocele and inguinal pain. All patients underwent surgery, mostly with orchi-funicolectomy. Adjuvant therapy was administered to 15 patients (20%). Overall median survival was 26.2 months (95% CI 22.1–52.1); 3-, 5- and 10-year survival was 49%, 30% and 18%. Older age at diagnosis and presence of distant metastasis (HR 1.91, CI: 0.85–4.26) were negative prognostic factors. Adjuvant therapy was associated with higher mortality (HR 2.54, CI: 1.25–5.15), indicating a more advanced stage at diagnosis. Conclusions: Surgery remains the mainstay of treatment for MTVT; adjuvant therapy in our study did not improve outcome. Data from cancer registries are essential for rare cancers, but they should be integrated routinely with additional diagnostic and therapeutic information.

## 1. Introduction

In 2009, the International Agency for Research on Cancer (IARC) identified additional asbestos-related tumor sites: to lung cancer and mesothelioma of all sites, laryngeal and ovarian cancers have been added [1].

Mesothelioma of the tunica vaginalis testis (MTVT) is an extremely rare cancer arising from the serosal membrane lining the cavities of the scrotum, and accounting for less than 1% of all mesotheliomas [2,3,4,5]. The SEER (Surveillance, Epidemiology and End Results) Program from the US National Cancer Institute estimated for MTVT a mean annual standardized incidence rate in 1973–2013 of 0.054 per million person-years [6]. In Italy, in the 1993–2015 period, the mean standardized (world standard population as reference) incidence rate of the disease was 0.095 per million person-years [7]. Asbestos is an established risk factor; in a recent study on 80 patients, occupational exposure to asbestos was found for 47 (59%) cases and associated with a three-fold increased risk of MTVT [7]. Trauma, prolonged inflammation and recurrent hydrocele have also been correlated with the development of the disease [8].

Due to the rarity of MTVT, only case reports and small case series have been published so far [9,10], hampering the development of new knowledge and expertise, and particularly of a recognized diagnostic and therapeutic algorithm. Clinical presentation of MTVT is unspecific; the most frequent signs and symptoms at the onset are a testicular/scrotal mass or swelling, hydrocele and pain [2,3,4]. A timely, extensive surgical treatment is currently considered the best option of care [11,12], but the role of adjuvant treatments (including local radiotherapy and/or chemotherapy or other systemic treatments) remains undefined [2,3,4]. Preoperative imaging with scrotal ultrasonography and abdominal CT scan may reveal subtle anomalies in the tunica surface or more overt soft-tissue masses [2,3,4]. However, preoperative diagnosis can be challenging, and most cases are diagnosed incidentally during surgery or after the pathological examination [3]. Only a few cases of metastatic MTVT have been reported. The benefit of systemic treatments in these patients is unclear, as they were excluded from the main trials in mesothelioma [13,14,15], which have established the current standard therapy of unresectable pleural mesothelioma.

Rare cancers pose challenges for uncertainty of diagnosis, lack of established therapies, poor research opportunities and difficulties in clinical trials [16]. For these reasons, patients with rare tumors frequently face delays in diagnosis and care, and receive suboptimal treatment [17]. For these cancers, centralized data collection by population-based registries remains fundamental in reporting patient care and outcomes in the real world. Here, we report the results of an analysis of clinical data of a large series of MTVT patients, systematically recorded by the Italian National Mesothelioma Registry (ReNaM, Registro Nazionale Mesoteliomi) from 1994 to 2021. This study aimed to review the diagnostic and therapeutic management of these patients, with the ultimate goal to improve the knowledge on natural history and clinical approach to this rare cancer.

## 2. Materials and Methods

### 2.1. Study Design, Participants and Data Sources

This study is based on data extracted from the Italian Mesothelioma Registry (Registro Nazionale Mesoteliomi, ReNaM) dataset. ReNaM is a population-based registry that collects information on individuals with mesothelioma (any site: pleura, peritoneum, pericardium and tunica vaginalis testis) from all regions in Italy [18]. It is organized as a network of 21 regional centers (Centri Operativi Regionali, COR), which periodically send information to the ReNaM; some regions started collecting mesothelioma cases in 1993, others started later [18]. Reporting of mesothelioma cases to CORs is compulsory by law (277/1991 and 81/2008) [18]. However, to ensure complete reporting, an active search of mesothelioma cases is routinely performed by exploiting several sources, including hospital discharge records and mortality datasets. Hospital medical records regarding mesothelioma are reviewed by each COR. Patients or their next-of-kin are interviewed using a standardized questionnaire by qualified personnel to investigate lifetime occupational and non-occupational asbestos exposure. Asbestos exposure is evaluated and classified according to ReNaM guidelines as: occupational, non-occupational (including familial, environmental and leisure-related) and not exposed [18].

### 2.2. Variables and Outcomes

For each case of MTVT, the following variables are routinely recorded by ReNaM: year of diagnosis, patient age at diagnosis, histological subtype (epithelioid, biphasic, sarcomatoid), source of asbestos exposure and modality of exposure evaluation (with direct or indirect interview). Additional clinical information include clinical presentation, presence of distant metastases at diagnosis and therapeutic management, including the type of surgical intervention and adjuvant therapies (chemotherapy and/or loco-regional radiotherapy) administered.

For all patients, we ascertained vital status and date and cause of death information as of 31 December 2023.

### 2.3. Statistical Analysis

A descriptive analysis of patient characteristics was performed. We calculated crude and standardized rates for the period 2000–2021 using the standard European (2013) and world (Segi’s) populations. Patients with no treatment information were excluded from further analyses. Overall survival was measured from the date of diagnosis to the date of death from any cause. Follow-up time was truncated at 10 years after the diagnosis. To evaluate potential risk factors, we performed Kaplan–Meier analysis and fitted multivariate Cox regression models to calculate hazard ratios (HR) and 95% confidence intervals (CI) for selected variables, including age at diagnosis (<65, 65–74, and ≥75 years), period of diagnosis (1994–1998, 1999–2004, 2005–2010, 2011–2016, 2017–2021), histotype, presence of distant metastasis at diagnosis (no vs. yes) and treatment group (surgery only or surgery plus any adjuvant therapy). All statistical analyses were performed using Stata 18, StataCorp, TX, USA [19].

## 3. Results

Between January 1994 and December 2021, 104 cases of MTVT were registered by ReNaM. The crude rate was 0.17 per million person-years, and rates standardized based on European and world populations were 0.18 and 0.08, respectively. Patient baseline characteristics are shown in Table 1. Overall, median age was 72 years (range 17–92), with nearly 70% of patients older than 65 years. Epithelioid histotype was the most frequent, being diagnosed in 54 (52%) cases in the whole population. Nearly half of patients received a direct interview for exposure evaluation. Previous asbestos exposure, almost exclusively occupational, was identified in two thirds of evaluated cases. Among unexposed cases, no clusters of residents in geographical areas known for the presence of naturally occurring asbestos were found. Median latency (years between first exposure and diagnosis) was 54 years (13–75) in occupationally exposed cases. In patients with other sources of exposure, latency was between 30 to 60 years.

Complete data on therapeutic management were available for 74 cases (71%); no differences were observed between this subgroup and the whole case list regarding age, histological subtype and asbestos exposure.

Table 2 summarizes the clinical characteristics of these 74 patients. The most frequent presentation was a scrotal or testicular swelling or mass, observed in 52 of 74 cases (70%), followed by the presence of hydrocele (46 patients, 62%). Scrotal or inguinal pain was reported in a minority of cases (17 patients, 23%). Unexpectedly, imaging evaluation, including, in most patients, scrotal and abdominal ultrasonography and chest and abdominal computed tomography scans, detected distant metastasis at diagnosis in one out seven patients (14%). In the majority of cases (70%), metastasis site was the lung. Only 19 patients (26%) had a pre-operative histological diagnosis of mesothelioma.

All patients underwent surgery (Table 3), mostly with orchi-funicolectomy (58 cases, 78%). A few patients (7, 10%) were treated with a more extended surgery including orchi-funicolectomy, hemiscrotectomy and homolateral inguinal lymphadenectomy. Eight patients (11%) underwent minor surgical procedures, while for one patient the extent of surgical resection was not reported (Table 3). Adjuvant therapy was administered to 15 patients (20%); 9 received chemotherapy alone, 2 loco-regional radiotherapy on scrotal and homolateral inguinal areas and 4 received both treatments. Chemotherapy was platinum-based in the majority of cases.

**Figure 1:**
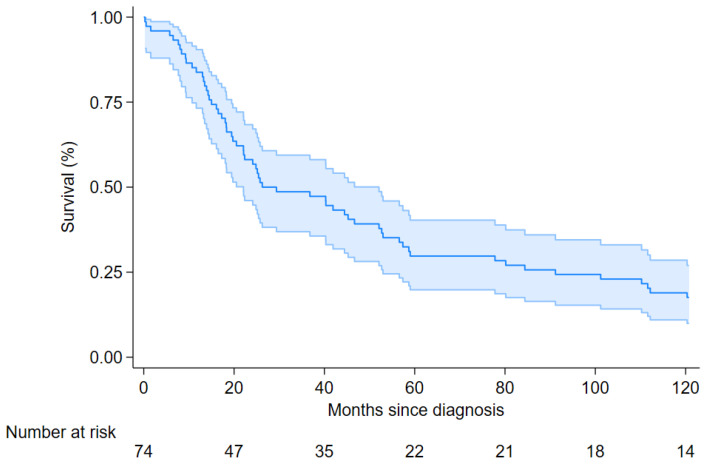
Ten-year overall survival for patients with MTVT, Italy, 1994–2021. Cases with treatment information (n = 74).

Overall median survival of the 74 patients was 26.2 months (95% CI 22.1–52.1), Figure 1); 3 yr, 5 yr and 10 yr survival rates were 49%, 30% and 18%, respectively.

**Figure 2:**
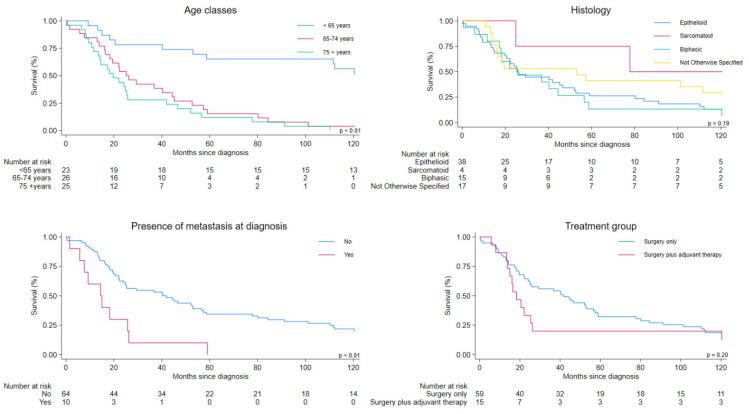
Ten-year overall survival according to selected risk factors for patients with MTVT, Italy, 1994–2021. Cases with treatment information (n = 74).

Table 4 shows survival in prespecified patient groups. Patients aged 65 years or older had strongly worse survival (Figure 2, Age classes). The presence of distant metastasis at diagnosis was associated with increased mortality (Figure 2, Histology). No important differences in survival were observed according to histological subtype (Figure 2, Presence of metastasis at diagnosis). Patients receiving adjuvant therapy had more than twofold increased risk of death (median survival 18.3 months vs. 41.9 months), (Figure 2, Treatment group).

## 4. Discussion

Our study confirmed the extreme rarity of MTVT, with 104 cases registered on a national basis in a 28-year span. Similarly to other more common sites of mesothelioma [20], MTVT was a disease of the elderly, with a median age of 72 years at diagnosis. Most patients had epithelioid histology, and in the majority of cases, a history of occupational asbestos exposure was reported. Surgery was the mainstay of treatment in all cases, with only a minority of patients receiving adjuvant chemotherapy and/or radiotherapy, with no improvement in outcome. Age older than 65 years, presence of distant metastasis at diagnosis and administration of adjuvant therapy were negative prognostic factors.

One of the major difficulties in managing MTVT is the lack of an accurate preoperative diagnosis. In accordance with previously published data [2,3,4], histological diagnosis in our cases was mostly achieved during or after primary surgery. Clinical presentation was consistent with previous series or reviews, with scrotal or testicular swelling and the presence of a hydrocele, often with local inflammation [2,3,4,9,10]. Notably, the rapid growth or recurrence of a hydrocele (particularly when hemorrhagic) and the presence of a scrotal/testicular mass should be suspicious for the diagnosis of MTVT, and should prompt imaging with ultrasonography and abdominal CT scan. In these cases, serum tumor markers can help in ruling out germ cell testicular cancer [21].

Surgery is the mainstay of treatment of MTVT [11,12]. In our series, all patients underwent intervention with the aim of macroscopic removal of the tumor. Most patients were treated with major surgery comprising orchi-funicolectomy, with hemiscrotectomy and/or inguinal lymphadenectomy in a minority of cases. Although it is difficult, based on literature data, to establish the optimal extent of surgery and, in particular, the benefit of adding hemiscrotectomy and lymphadenectomy of inguinal or even retroperitoneal nodes [4,22], it seems reasonable to achieve the maximum possible cytoreduction with surgery. For men with an incidental diagnosis of MTVT during hydrocelectomy, a timely completion of a hemiscrotectomy with en bloc orchi-funicolectomy is recommended, due to potential tumor seeding [4].

Adjuvant treatments after surgery were administered in a minority of patients (15 of 74, 20%), with heterogeneous regimens, which mainly comprised platinum-based chemotherapy, postoperative radiotherapy or both. The delivery of post-operative treatments was detrimental for survival, indicating confounding by indication (adjuvant treatments performed in patients with larger tumors with higher risk of recurrence) [23]. Similarly, in a review of 289 published cases of MTVT, 49 patients (17%) only received adjuvant therapy, with no improvement in survival as compared to patients treated with surgery alone; median survival was 24 months in both groups [2]. Due to the limited number of cases receiving post-operative treatments, and the lack of prospective or randomized data, the role of a multimodality approach in MTVT remains undefined.

Age older than 65 years and stage were negative prognostic factors. Similar results were reported in large case reviews [3]. Unexpectedly, metastases were detected in 14% of patients, with lung as the most frequent site, suggesting a mainly hematogenous pattern of disease spread.

Epithelioid histology was the most commonly found variety, similarly to what was reported in all major published series [2,3,4]. However, the favorable prognostic role of epithelioid subtype [2,24] was not confirmed in our series, likely due to the small number of non-epithelioid tumors, particularly of the sarcomatoid variant. The immunohistochemical profile of MTVT is similar to that of pleural mesothelioma, including markers such as calretinin, cytokeratin 5/6 and WT-1 [25]. MTVT exhibits a mutational profile similar to that of the pleura and peritoneum; however, alterations in CDKN2A and BAP1 (BRCA1-associated protein 1) are less common. Therefore, BAP1 and MTAP (methylthioadenosine phosphorylase) expression may be lost [26], but their sensitivities in MTVT appear to be lower. Histologic features, including architectural patterns and nuclear grade, have prognostic significance in pleural mesothelioma, although their correlation with genetic alterations has not been well studied yet [27]. Data on this correlation are lacking in MTVT, however the integration of molecular markers with histological features has the potential to improve its diagnosis and therapeutic management.

Finally, MTVT should be differentiated from exceedingly rare entities such as mesothelioma of uncertain malignant potential (MUMP) and well-differentiated papillary mesothelial tumor (WDPMT) of the tunica vaginalis testis, which are characterized by a benign course if completely resected [28].

## 5. Conclusions

In summary, to our knowledge, this is the largest population-based study on MTVT, including clinical details on diagnosis and treatment. It has the strength of a national surveillance evaluation of a very rare cancer, with strict inclusion criteria (cases were included only after review of all medical records, including imaging and cyto-histological data), and with thorough assessment of asbestos exposure made by experienced personnel using a standardized structured questionnaire. On the other hand, the study has the limitation of sometimes incomplete clinical data and of the lack of specific data quality evaluation. In particular, the registry does not routinely collect data on clinical presentation, tumor size, T-stage (including metastasis at presentation) or extent of surgical resection, which are risk factors for mortality. This information is almost complete for the regions in Northern Italy, in which most patients (72 out of 104) were residents. Therefore, although there is the potential for selection bias, we think our findings are fairly representative of clinical management of MTVT in our country.

Data provided by dedicated cancer registries remain essential, but their quality has to improve, especially on clinical presentation and therapeutic information that needs to be collected routinely. The integration of population-based registries with clinical national and international networks [16,17,18,27] and the implementation of prospective observational studies with detailed clinical and treatment information will offer the opportunity to improve knowledge, centralization of treatment, clinician expertise and, ultimately, the quality of patient management.

## Figures and Tables

**Table 1:** Characteristics of patients with MTVT, Italy, 1994–2021.

Variable	Total Cases N (%)	Cases with Treatment Information N (%)	Cases Without Treatment Information N (%)	*p*-Value ^a^
**Total**	104 (100)	74 (100)	30 (100)	
**Age at diagnosis median (range)**	72 (17–92)	72 (17–89)	76 (40–92)	0.16
**Age at diagnosis (years)**				
<45	9 (9)	8 (11)	1 (3)	0.13
45–64	21 (20)	15 (20)	6 (20)	
65–74	32 (31)	26 (35)	6 (20)	
≥75	42 (40)	25 (34)	17 (57)	
**Period of diagnosis**				
1994–1998	10 (10)	10 (14)	0	0.04
1999–2004	23 (22)	18 (24)	5 (17)	
2005–2010	24 (23)	17 (23)	7 (23)	
2011–2016	28 (27)	20 (27)	8 (27)	
2017–2021	19 (18)	9 (12)	10 (33)	
**Morphology (ICD-O-3 code)**				
Epithelioid (90523)	54 (52)	38 (51)	16 (53)	0.34
Biphasic (90533)	17 (16)	15 (20)	2 (7)	
Sarcomatoid (90513)	6 (6)	4 (5)	2 (7)	
Not otherwise specified (90503)	27 (26)	17 (23)	10 (33)	
**Exposure evaluation**				
Direct interview	58 (56)	43 (58)	15 (50)	0.65
Indirect interview	30 (29)	21 (28)	9 (30)	
None	16 (15)	10 (14)	6 (20)	
**Sources of asbestos exposure ^b^**				
Occupational	56 (64)	40 (63)	16 (67)	0.46
Non-occupational	3 (3)	2 (3)	1 (4)	
Familial	1 (1)	1 (2)	1 (4)	
Environmental	1 (1)	0	0	
Leisure-related	1 (1)	1 (2)	1 (4)	
Unexposed	29 (33)	22 (34)	7 (29)	
**Latency (years between first exposure and diagnosis—median (range))**				
Occupational	54 (13–75)	52 (13–75)	54 (24–70)	0.33
Non-occupational (years)				
Familial		51		
Environmental			59	
Leisure-related		32		

^a^ From a chi-squared test, except for age (Wilcoxon rank-sum test). ^b^ Only for cases with exposure evaluation by direct or indirect interview. Abbreviations: MTVT, mesothelioma of tunica vaginalis testis; ICD-O-3, International Classification of Diseases for Oncology, Third Edition.

**Table 2:** Clinical and radiological presentation of patients with MTVT, Italy, 1994–2021. Cases with treatment information (n = 74).

Variable	N	%
**Total**	**74**	**100**
**Age at diagnosis**, **median (range)**	72 (17–89)	
**Clinical presentation**		
Scrotal or testicular swelling/mass	52	70
Hydrocele or hemorrhagic hydrocele	46	62
Epididymitis/orchitis/other local inflammations ^a^	11	15
Scrotal or inguinal hernia	12	16
Scrotal or inguinal pain	17	23
Other ^b^	7	10
**Distant Metastasis at diagnosis ^c^**		
No	64	87
Yes	10	14

^a^ Including: 2 pachyvaginalitis and 1 scrotal abscess. ^b^ Including: 1 spermatic cord torsion, 2 epididymal cysts, 1 weight loss and 3 incidental findings. ^c^ As assessed by imaging evaluation. Metastasis site: 7 lungs, 3 other sites. Abbreviations: MTVT, mesothelioma of tunica vaginalis testis.

**Table 3:** Upfront therapeutic management of patients with MTVT, Italy, 1994–2021. Cases with treatment information (n = 74).

Variable	N	%
**Total**	**74**	**100**
**Surgery**	74	100
Orchi-funicolectomy	58	78
Orchi-funicolectomy + hemiscrotectomy and/or inguinal lymphadenectomy	7	10
Subtotal surgery ^a^	8	11
Unknown	1	1
**Surgery + adjuvant treatment(s)**		
Any adjuvant treatment	15	20
Adjuvant Radiotherapy	2	3
Adjuvant Chemotherapy	9	12
Both	4	5

^a^ Including removal of the tumor mass in 6 cases, hydrocelectomy in 1 case, biopsy only in 1 case. Abbreviations: MTVT, mesothelioma of tunica vaginalis testis.

**Table 4:** Overall survival and hazard ratios (HR) of deaths according to selected risk factors for patients with MTVT, Italy, 1994–2021. Cases with treatment information (n = 74).

Variable	N	Deaths N (%)	Overall Survival, (Months) Median	Crude HR	95% CI	Adjusted HR	95% CI
**Overall**	74	61 (82)	26.2 (22.1–52.1)				
**Age at diagnosis (years)**							
<65	23	11 (48)	NC (NC- 53.0)	1.00	Reference	1.00	Reference
65–74	26	25 (96)	25.2 (16.5–44.5)	4.97	2.32–10.64	6.69	2.77–16.14
≥75	25	25 (100)	19.7 (13.5–25.8)	6.70	3.07–14.60	10.50	4.16–26.50
**Period of diagnosis**							
1994–1998	10	8 (80)	13.3 (0.3–59.0)	1.00	Reference	1.00	Reference
1999–2004	18	13 (72)	25.4 (15.0–110.2)	0.65	0.27–1.57	0.54	0.19–1.52
2005–2010	17	11 (65)	41.9 (19.7 -NC)	0.49	0.20–1.21	0.46	0.17–1.23
2011–2016	20	20 (100)	22.3 (14.5–80.2)	0.97	0.43–2.21	0.69	0.27–1.76
2017–2021	9	9 (100)	40.3 (11.6–53.0)	1.13	0.43–2.97	0.96	0.33–2.76
**Morphology**							
Epithelioid (90523)	38	33 (87)	25.4 (19.7–46.7)	1.00	Reference	1.00	Reference
Biphasic (90533)	15	14 (93)	25.7 (11.6–44.5)	1.18	0.63–2.21	1.72	0.85–3.45
Sarcomatoid (90513)	4	2 (50)	77.8 (25.9-NC)	0.33	0.08–1.39	0.36	0.08–1.59
NOS (90503)	17	12 (71)	53.0 (14.2-NC)	0.65	0.34–1.27	1.25	0.60–2.61
**Presence of metastasis at diagnosis**							
No	64	51 (78)	40.3 (22.3–56.6)	1.00	Reference	1.00	Reference
Yes	10	10 (100)	14.5 (1.6–25.8)	3.09	1.53–6.24	1.91	0.85–4.26
**Treatment group**							
Surgery only	59	48 (81)	41.9 (24.1–56.6)	1.00	Reference	1.00	Reference
Surgery plus adjuvant therapy	15	13 (87)	18.3 (13.5–25.4)	1.50	1.53–6.24	2.54	1.25–5.15

Abbreviations: MTVT, mesothelioma of tunica vaginalis testis; CI, confidence intervals; NOS, not otherwise specified; NC, not calculable.

## Data Availability

Data presented in this study are available on request from the corresponding author.
